# Virtual Reality in Awake brain Surgery (VIRAS) stage I: Proof of concept and tolerance validation during scheduled orthopedic surgery

**DOI:** 10.1371/journal.pone.0329894

**Published:** 2025-09-03

**Authors:** Vanessa Saliou, Guillaume Dardenne, Celine Panheleux, Florence Le Vourc’h, Justine Bleunven, Ilias Maoudj, Basile Longo, Frederic Dubrana, Agathe Yvinou, Marie Fernandez, Maelys Consigny, Emmanuel Nowak, Dewi Guellec, Romuald Seizeur

**Affiliations:** 1 Service des Explorations Fonctionnelles Neurologiques, CHU de BREST, Cavale Blanche, Brest, France; 2 LaTIM – Laboratoire de Traitement de l’Information Médicale, UMR INSERM 1101, Hôpital Morvan, Brest, France; 3 Service de Neurochirurgie, CHU de BREST, Cavale Blanche, Brest, France; 4 Service de Chirurgie Orthopédique, CHU de BREST, Cavale Blanche, Brest, France; 5 Faculté de Médecine et Sciences de la santé. 22, Université de Bretagne Occidentale (UBO), Brest, France; 6 Direction de la Recherche Clinique et de l’Innovation, CHU de BREST, Morvan, Brest, France; 7 Centre d’Investigations et de Recherche Clinique, CHU de BREST, Cavale Blanche, Brest, France; Johns Hopkins University, UNITED STATES OF AMERICA

## Abstract

**Introduction:**

The VIRAS (Virtual Reality in Awake Surgery) project is a two-stage, adaptive study. Its goal is to demonstrate the tolerance of the virtual reality (VR) headset for performing cognitive neuro-monitoring during awake brain surgery. Awake surgery involves operating on patients who remain conscious during the procedure and is most commonly employed in interventions such as tumor resections and epilepsy treatments. This approach allows surgeons to monitor and preserve critical brain functions by engaging the patient in real-time assessments of motor, sensory, and cognitive capabilities. The use of immersive distractions such as VR can help reduce anxiety and discomfort during awake craniotomy. We present the results of the first stage of the study, conducted in patients undergoing scheduled orthopedic surgery under regional anesthesia, aimed at validating the tolerance and safety of using the VR headset in the operating room.

**Materials and methods:**

Eligibility required a minimum predicted surgery duration of one hour. All participants received standardized training in the use of VR headset the day before surgery. Investigators supervised intraoperative neurofunctional testing delivered through the VR system. Tolerance and safety were evaluated using VAS scores, the Simulator Sickness Questionnaire (SSQ), and the State-Trait Anxiety Inventory (STAI). Acceptability was assessed among healthcare providers. The primary outcome was defined as successful maintenance of the VR headset and completion of neurofunctional testing for at least one hour. Data analysis employed the Sequential Probability Ratio Test (SPRT) with predefined thresholds (P₀ = 0.6, P₁ = 0.8; N_min_ = 10, N_max_ = 50).

**Result:**

The first 10 patients completed the procedure successfully, meeting the primary endpoint and leading to early study termination per SPRT design. The VR headset was well tolerated in all cases, with no adverse events reported. Median VAS tolerance scores were high (training: 9.0; intraoperative: 10.0). SSQ scores indicated minimal cybersickness. All participants completed neurofunctional tests during surgery and expressed willingness to reuse the device. Acceptance among healthcare providers was excellent (median VAS: 10).

**Conclusion:**

The initial phase of the VIRAS study demonstrated excellent overall tolerance of the VR headset by both participants and the healthcare professionals involved in orthopedic surgery.

## Introduction

Awake surgery is an innovative neurosurgical approach that enables maximal precision in lesion removal while preserving essential brain functions [1]. Endorsed by the European Association of Neuro-Oncology, this technique is currently the preferred approach for resecting low-grade glial tumors [2–5]. By enabling surgeons to operate while the patient is conscious, it ensures preservation of cognitive and neurological abilities, thereby enhancing patient outcomes and quality of life [1,6]. Awake surgery allows for the establishment of individualized brain functional mapping. To delineate the excision area, the neurosurgeon utilizes the technique known as direct electrical stimulation (DES) [7,8]. This brief electrical discharge, lasting for 4 seconds and shorter than epileptic time, reversibly disrupts function, which is simultaneously tested by either the neurologist, neuropsychologist or speech therapist using specific tests [1,9]. The patient is instructed to perform cognitive tasks to identify functional areas inhibited by electrical stimulation. Intraoperatively, tasks are presented on a computer screen or paper. Each cortical site is stimulated multiple times at varying intensities to confirm functional involvement. The type of disruption is classified by the neuropsychologist or speech therapist who conducted the preoperative assessments and is present in the operating room [1,6,7].

Despite ongoing advances in functional imaging, intraoperative mapping remains essential to accurately establish an individual’s functional brain organization [1,10,11]. Functional MRI (fMRI) detects brain activity by monitoring real-time changes in blood oxygenation and local cerebral blood flow. Increased neuronal activity in a specific brain region leads to elevated oxygen consumption, triggering a corresponding rise in blood flow to meet metabolic demands. However, the main limitation of fMRI is its limited spatial specificity, which complicates interpretation. Activated regions may overlap across different tasks or emotional states, making it difficult to accurately localize specific functions or assess individual patient conditions [1,12,13]. Tools for language assessment during awake surgeries are now well established [14,15]. However, few studies have addressed the evaluation of other cognitive domains, such as executive functions, visuospatial and social cognition, or the study of visual fields. This is particularly due to the challenges of adapting standard tests to the constraints of the intraoperative setting [16,17]. Assessing 3D visualization abilities, lateral visual field perception, and visuospatial hemineglect remains particularly challenging with current methods, such as paper-and-pencil tasks or 2D screen-based assessments. The use of a virtual reality (VR) headset could significantly enhance the development of more sensitive tools for the early detection of functional visual impairments. In addition, VR enables multimodal testing through realistic simulations of daily life, including activities related to personal care, work, leisure, sports, or musical performance, within an immersive environment [16,17]. Yet, these cognitive functions are just as essential as language for maintaining individual autonomy. Despite recommendations from the European Low-Grade Glioma Network (ELGGN), neurofunctional testing practices remain heterogeneous across center, with no universal standardization to date [6].

At our institution, neurofunctional testing during awake surgery is currently conducted using a two-dimensional screen to display PowerPoint presentations assessing language, memory, sensory, motor, or visual functions, depending on tumor location. This approach enables personalized, standardized testing tailored to both the lesion site and the patient’s abilities. The integration of VR as a tool for delivering individualized neurofunctional assessments represents a highly promising direction, already explored by several authors [16–18]. Furthermore, the use of software informed by current connectome research [19] to guide multimodal cognitive testing could ultimately facilitate the harmonization of practices [2,6] and contribute to making awake neurosurgery more accessible.

The ideal VR tool in this context should be well tolerated by patients and enable the implementation of neurofunctional tests tailored to each patient’s specific profile, without adding constraints for the healthcare professionals involved in these complex surgical procedures [20]. In 2019, an observational ergonomic study was conducted at Brest University Hospital to evaluate the workflow of awake surgery and explore the potential benefits of using a virtual reality headset in this setting. By isolating the patient from the operating room environment, the device was hypothesized to improve the quality of neurofunctional testing by enhancing patient focus and reducing anxiety. It may also improve ergonomic comfort for both the patient and the monitoring team by reducing equipment clutter near the patient, as the headset replaces the need for a remote screen. Several studies have reported that patients may experience discomfort, anxiety, or pain during awake craniotomy procedures [21–23]. The use of VR may help optimize neurofunctional testing performance while also reducing the anxiety and discomfort associated with being awake in the operating room. Increasing evidence suggests that virtual reality can be beneficial in addressing these challenges [18,24].

Existing scientific literature, as well as the VR headset user manual, has not identified any significant or life-threatening risks associated with its use in healthcare settings. However, certain minor side effects under normal conditions—such as nausea or vomiting—could pose greater concerns in the operating room. The potential risk of seizures, particularly relevant in patients with brain lesions, warrants careful evaluation. Although current studies on the use of VR in awake neurosurgery are reassuring regarding this issue, larger-scale research is needed to confirm its safety profile. [18,25]. Epileptic risk is managed in accordance with current guidelines, using intravenous antiepileptic drugs with rapid onset of action, minimal cognitive side effects, and no enzymatic induction. Risk assessment includes a preoperative electroencephalogram and neurological evaluations conducted both before and after surgery [26].

The VIRAS (Virtual Reality in Awake Surgery) project is a two-stage, adaptive, single-arm study. Its ultimate goal is to demonstrate that the tolerance of the virtual reality mask for performing neuro-monitoring during awake brain surgery is sufficient to validate its implementation for routine care. Here, we present the results of the first stage of the VIRAS project, conducted on patients who volunteered to undergo neurofunctional tests via the VR headset during scheduled orthopedic surgery. The success of this first part of the VIRAS project, according to a strictly pre-specified rule relying on acceptance test, was considered as necessary for the continuation of the project and the opening of inclusions to patients undergoing awake brain surgery. Indeed, it was considered important to first confirm the tolerance of such management in the general context of surgical care before proceeding with its validation in the critical population of patients undergoing awake brain surgery [24,27,28]. The primary objective of this study was therefore to determine the tolerance of the device during the surgery, performed under regional anesthesia. Secondary objectives were to describe adverse events related to the use of the VR headset, including those necessitating its removal, as well as to determine the acceptability of the device by both the patient and the surgical team in the operating room.

## Materials and methods

### Study design and participants

In this first stage of the VIRAS project, participation in the study was offered to consecutive adult patients (aged 18–75 years old) who presented to orthopedic or anesthesia consultations for scheduled orthopedic surgery, performed under regional anesthesia. An additional selection criterion was a predicted duration of surgery of at least one hour, in order to assess the tolerance of the virtual reality mask over a sufficient period of use. All orthopedic surgeons and anesthetists at Brest University Hospital (CHU) were informed about the study and had the opportunity to offer participation in the VIRAS study to their eligible patients. The main exclusion criteria were the following: 1. Known central neurological pathology/ cognitive disorders, 2. Abnormal MMSE (Mini-Mental State Examination) score (< 23 without a certificate of education, < 27 with a certificate of education), 3. History of vertigo, 4. Claustrophobia, 5. Visual impairment not compatible with the use of the device, 6. Outpatient surgical management. All participants underwent a standardized training session on the use of the VR headset under the supervision of a study investigator the day before surgery. The session included a one-hour video presentation of the main neuropsychological tasks used in awake neurosurgery, displayed within a virtual movie theater. On the day of surgery, the VR device was installed in the operating room by a study investigator, who also oversaw its monitoring throughout the surgical procedures. Standard surgical protocols remained unchanged. Patient enrollment took place between May 24, 2022, and December 19, 2023. All participants provided written informed consent prior to inclusion. This study was approved by the appropriate ethics committee (“*Comité de Protection des Personnes Nord-Ouest II”*) on April 19, 2021, and was registered on ClinicalTrials.gov (NCT05151822).

### Virtual reality mask and neurofunctional tests

The equipment used was an Oculus Quest 1 (64GB), employed without any modifications for use in the operating room during orthopedic surgery. The headset was positioned and securely fastened to the patient using the original head strap provided with the device. A Samsung Galaxy Tab S6 Android 9.0 (Pie) Tablet controlled administration of neurofunctional tests. The neurofunctional tests administered throughout the duration of the surgical intervention were those commonly used in routine care during awake brain surgeries conducted within our institution (S1 File). The tests were presented in a two-dimensional format within the three-dimensional virtual environment of the VR headset, using the SkyBox VR Video Player® software. It was planned that any adverse effects or abnormal sensations causing discomfort or pain reported by the patient during the procedures, which could impede the continuation of neurofunctional testing, would result in its immediate discontinuation. In the event of an intraoperative emergency (e.g., hemodynamic instability), the anesthesiologist and surgeon were authorized to interrupt the VR session at any time.

### Data collection and outcome measures

The following characteristics were collected at inclusion: age, gender, body mass index (BMI), medical history, allergies, current medications, substance use, handedness, eyeglasses worn and MMSE. The overall tolerance of the VR headset by the participants was assessed quantitatively using a 0–10 Visual Analog Scale (VAS) administered after the training session and immediately after the surgical intervention (alternatively the following day). Simulator Sickness Questionnaire (SSQ) was similarly administered, both after the training session and after surgery. The State-Trait Anxiety Inventory (STAI) was administered to patients after the surgery (immediately or alternatively the following day) for assessing state anxiety and trait anxiety related to VR headset utilization in the operating room. All adverse events occurring in the operating room were documented, with specific notation of those requiring removal of the VR headset. Acceptability of utilization was also systematically assessed among healthcare providers (surgeon, anesthesiologist, operating room nurse and anesthesia nurse), using a 0–10 VAS. The primary outcome measure, of success/failure type, was the maintenance of the VR headset and the ability to conduct neurofunctional tests throughout the duration of the surgery (for at least one hour).

### Statistical analysis

Primary outcome was analyzed through sequential analyses, using the truncated binomial Sequential Probability Ratio Test (SPRT). This method allows for determining, after each new observation (success or failure), either the continuation of inclusions, the study’s termination for futility, or the validation of the device’s tolerance, according to pre-specified parameters [29,30]. The following parameters were used: P_0_ (low tolerance proportion below which the device would not be considered “tolerable”) = 0.6; P_1_ (high tolerance proportion above which the virtual reality mask would be considered “tolerable”) = 0.8; Nmin (minimum sample size) = 10; Nmax (maximum sample size) = 50. Table 1 summarizes the decision-making process following each new observation, starting from the 10th patient, based on these parameters and considering an alpha risk of 5% and a power of 80%. The data are presented as the median and interquartile range (IQR) for quantitative variables and as frequency and percentage for qualitative variables. The statistical analyses were conducted using the SAS software version 9.4.

**Table 1:** Decision-making process at each new observation from the 10th participant, according to the truncated binomial Sequential Probability Ratio Test.

Participant rank	Stop for futility if the number of successes is less than or equal to:	Stop for efficacy if the number of successes is equal to or greater than:
10	5	10
11	6	11
12	6	12
13	7	13
14	8	13
15	9	14
16	9	15
17	10	15
18	11	16
19	11	17
20	12	17
21	13	18
22	13	19
23	14	20
24	15	20
25	16	21
26	16	22
27	17	22
28	18	23
29	18	24
30	19	25
31	20	25
32	21	26
33	21	27
34	22	27
35	23	28
36	23	29
37	24	29
38	25	30
39	25	31
40	26	32
41	27	32
42	28	33
43	28	34
44	29	34
45	30	35
46	30	36
47	31	37
48	32	37
49	33	38
50	33	39

The following parameters were used: P_0_ (low tolerance proportion below which the device would not be considered “tolerable”) = 0.6; P_1_ (high tolerance proportion above which the VR mask would be considered “tolerable”) = 0.8; Nmin (minimum sample size) = 10; Nmax (maximum sample size) = 50.

## Results

The first 10 patients completed the procedure successfully, meeting the primary endpoint and leading to early study termination per SPRT design.

### Study population (Fig 1; Table 2)

Exhaustive study poplulation’s characteristics are provided in Table 2. Among the 10 participants, 7 (70.0%) were women. Median age was 68.5 years (IQR: 60.0–72.0), with a minimum of 55 and a maximum of 75 years. 7 participants (70.0%) had an educational level equivalent to or higher than a high school diploma. 9 (90.0%) were right-handed. All participants reported regular wearing of prescription glasses. 8 (80.0%) participants reported daily intake of medication, including 1 taking Duloxetine, 1 Lorazepam, 1 Tramadol and 1 Venlafaxine.

**Table 2:** Characteristics of the study population.

Patients characteristics		Total (n = 10)
Age, years	n (n missing)	10 (0 missing)
	Mean + /- SD	66.70 + /- 6.70
	Median (IQR)	68.5 (60.0–72.0)
	Range	55–75
Sex, n (%)	Male	3 (30.0%)
	Female	7 (70.0%)
BMI	n (n missing)	10 (0 missing)
	Mean + /- SD	30.87 + /- 9.99
	Median (IQR)	29.1 (24.9–31.9)
	Range	20–56
Educational level, n (%)	Below High school diploma	3 (30.0%)
	High school diploma	5 (50.0%)
	Bachelor’s degree	1 (10.0%)
	Master’s degree	1 (10.0%)
None	No	3 (30.0%)
	Yes	7 (70.0%)
Sport	*Missing*	7 (70.0%)
	No	2 (66.7%)
	Yes	1 (33.3%)
Music	*Missing*	7 (70.0%)
	No	1 (33.3%)
	Yes	2 (66.7%)
Other artistic activity, n (%)	*Missing*	7 (70.0%)
	No	3 (100.0%)
Smoking status, n (%)	Active smoker	1 (10.0%)
	Former smoker	6 (60.0%)
	Never smoked	3 (30.0%)
Alcohol use, n (%)	No	6 (60.0%)
	Yes	4 (40.0%)
Other substance use, n (%)	No	10 (100.0%)
Handedness, n (%)	Left-handed	1 (10.0%)
	Right-handed	9 (90.0%)
Prescription glasses, n (%)	Yes	10 (100.0%)
Currently on medication, n (%)	No	2 (20.0%)
	Yes	8 (80.0%)

BMI: Body Mass Index; IQR: Interquartile Range; SD: Standard Deviation.

**Fig 1:**
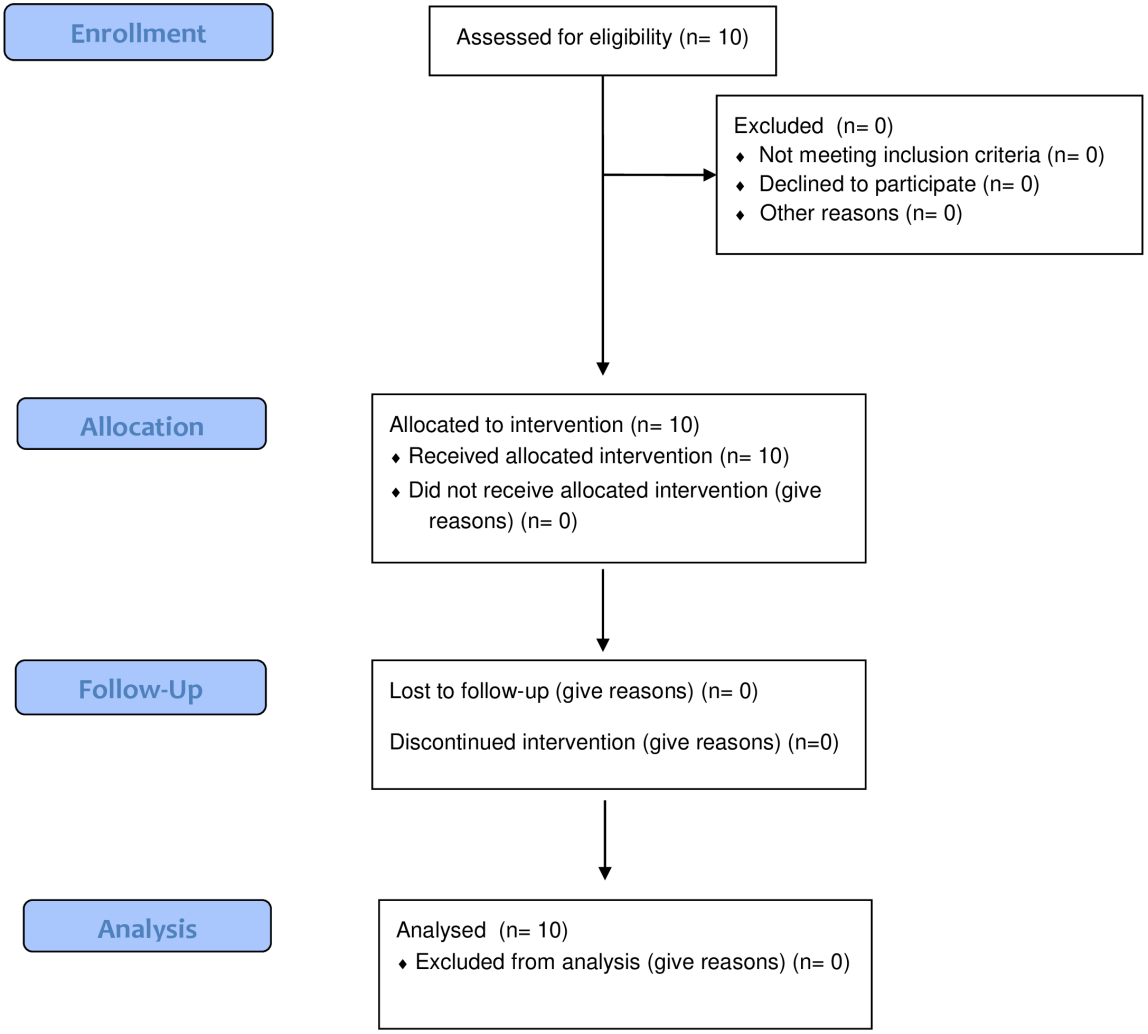
Flowchart.

### Virtual reality headset training session

All participants received a training session, with a median duration of 67.5 minutes (IQR: 65.0–70.0). The overall tolerance of the device was good, with a median VAS score of 9.0 (IQR: 9.0–10.0). The minimum value of the VAS score was 7.0. Detailed results of the SSQ administered after the training session are provided in Table 3. All reported cybersickness’s symptoms were rated as slight or negligible. Patients mainly described oculomotor symptoms. Notably, 6 participants reported “*fatigue*”, 6 reported “*eye strain*” 2 reported “*headache*” and 2 reported “*general discomfort*”.

**Table 3:** Simulator Sickness Questionnaire (SSQ) scores following VR training session.

		Total (n = 10)
Total SSQ score	n (n missing)	10 (0 missing)
	Mean + /- SD	3.20 + /- 3.12
	Median (IQR)	2.5 (1.0–4.0)
	Range	0–10
Cybersickness severity classification, n (%)	No symptoms	1 (10.0%)
	Moderate	1 (10.0%)
	Negligible	7 (70.0%)
	Slight	1 (10.0%)
Nausea sub-score	n (n missing)	10 (0 missing)
	Mean + /- SD	0.70 + /- 1.34
	Median (IQR)	0.0 (0.0–1.0)
	Range	0–4
Oculomotor sub-score	n (n missing)	10 (0 missing)
	Mean + /- SD	2.50 + /- 2.37
	Median (IQR)	1.5 (1.0–4.0)
	Range	0–7

IQR: Interquartile Range; SD: Standard Deviation.

### Virtual reality headset use in the operating room

The median duration of surgery was 67.0 minutes (IQR: 63.0–75.0). As previously noted, all participants tolerated the headset well and were able to complet neurofunctional testing throughout the entire surgical procedure. Overall tolerance of the device in the operating room was excellent, consistent with results from the training session, with a median VAS score of 10.0 (IQR: 9.0–10.0). No adverse events were reported during the scheduled surgeries. All participants indicated they would be willing to use the device again in a future procedure. Except for “*difficulty concentrating*”, SSQ scores were similar or improved compared to those observed after training (Table 4). Regarding anxiety levels, the median STAI “*trait*” score was 35.0 (IQR: 30.0–39.0) and the “*state*” score was 21.0 (IQR: 20.0–24.0). Acceptance among healthcare professionals was also excellent, with detailed results presented in Table 5.

**Table 4:** Simulator Sickness Questionnaire (SSQ) following the surgical intervention.

		Total (n = 10)
Total SSQ score	n (n missing)	10 (0 missing)
	Mean + /- SD	1.20 + /- 1.81
	Median (IQR)	0.5 (0.0–1.0)
	Range	0–5
Cybersickness severity classification, n (%)	No symptoms	5 (50.0%)
	Negligible	4 (40.0%)
	Slight	1 (10.0%)
Nausea sub-score	n (n missing)	10 (0 missing)
	Mean + /- SD	0.20 + /- 0.42
	Median (IQR)	0.0 (0.0–0.0)
	Range	0–1
Nausea sub-score	n (n missing)	10 (0 missing)
	Mean + /- SD	1.00 + /- 1.63
	Median (IQR)	0.0 (0.0–1.0)
	Range	0–4

IQR: Interquartile Range; SD: Standard Deviation.

**Table 5:** Acceptability of the VR headset in the operating room among healthcare professionals.

	Surgeons	Anesthesiologists	Operating room nurse	Anesthesia nurse
Median VAS score (IQR)	10 (10–10)	10 (10–10)	10 (8–10)	10 (8–10)
Minimum VAS score	8	8	7	7
Maximum VAS score	10	10	10	10

IQR: Interquartile Range; VAS: Visual Analog Scale.

## Discussion

The results of this initial phase of the VIRAS study demonstrate the favorable tolerance of the VR headset when used in the operating room to enable neuro-monitoring under optimal conditions. The primary outcome successful completion of neurofunctional testing throughout the full duration of scheduled orthopedic surgery was achieved in all participants. It is also noteworthy that all participants expressed willingness to undergo the same procedure in future surgical intervention, further supporting the overall tolerability of the device in a surgical context.

### Cybersickness

The SSQ was originally developed to detect cybersickness in military personnel trained on flight simulators individuals generally less susceptible to cybersickness than the general population [31]. As such, the scale offers high sensitivity for detecting cybersickness in clinical settings. In our study, no participant experienced more than slight symptoms on the SSQ, indicating excellent tolerance of the device in the operating room. The SSQ results obtained intraoperatively were fully satisfactory and consistent with those recorded during the training session the previous day, with the exception of reported difficulties in concentration, likely attributable to the specific context of the surgical procedure. While some studies involving virtual reality during awake craniotomy have reported symptoms of cybersickness, they did not employ standardized evaluation tools such as the SSQ, limiting the comparability and robustness of their findings [16–18].

### Anxiety

The STAI-Y (State-Trait Anxiety Inventory) includes two distinct subscales to assess anxiety: STAI-YA measures state anxiety (how the subject feels in a given moment), while STAI-YB assesses trait anxiety (general, long-term disposition toward anxiety). In our study, STAI results reflected excellent tolerance of the VR headset in the operating room. The median state anxiety (STAI-YA) score following the VR experience was 21.0, while the trait anxiety (STAI-YB) score was 35.0. These findings suggest that the use of a VR headset may help reduce intraoperative anxiety during awake procedures. In support of this hypothesis, several studies have explored the anxiolytic potential of VR in surgical settings. A prospective, single-center randomized controlled trial involving 50 patients undergoing joint replacement under regional anesthesia showed good tolerance of immersive VR, although it did not reduce overall sedation requirements [27]. Conversely, another randomized study found that VR immersion during hand surgery significantly reduced intraoperative propofol dosage and post-anesthesia care unit length of stay, without negatively affecting key patient-reported outcomes [28]. These mixed results underscore the need for further studies to clearly establish the benefits of VR in surgical contexts, particularly through the use of standardized protocols to assess both efficacy and acceptability in the operating room. To date, many studies have relied on indirect markers of tolerance such as pain intensity, intraoperative sedative use, or recovery time rather than validated psychological assessment tools. Standardized evaluation frameworks are therefore essential to objectively assess the contribution of VR to perioperative stress reduction and patient comfort [27,28].

If the use of a VR headset is expected to improve the prevention of neurofunctional deficits during awake craniotomy, it should also be considered a valuable tool for assessing and supporting mental health throughout the procedure. The risk of post-traumatic stress disorder (PTSD), first described by Milian et al. in a monocentric German study, must be recognized, anticipated, and managed when applicable. To date, no study has comprehensively evaluated stress, anxiety, and depression across all phases of awake brain surgery. In addition, psychiatric assessments vary considerably between centers, often relying on self-developed questionnaires whose reliability and validity remain uncertain [23].Among the validated tools, the STAI scale has already been used in several studies to evaluate anxiety and the psychological state of patients undergoing awake craniotomy [23,32–34], and could serve as a basis for standardization in future research.

### Healthcare providers’ tolerance

Regarding acceptability among healthcare professionals, it is noteworthy that the lowest VAS scores (7/10) were reported by paramedical staff, specifically the operating room nurse and the anesthesia nurse. Although overall satisfaction remained high, these findings highlight the importance of involving paramedical teams early in the implementation process and project planning to ensure optimal integration and acceptance.

### Limitations

This study presents several limitations. First, it was conducted at a single center, with a limited number of participants. Second, the sequential design inherently allows for early study termination upon validation of the tolerance hypothesis, which may result in a small final sample size. This can limit the generalizability of the findings, particularly with respect to the diversity of clinical scenarios and restrict the interpretation of certain secondary outcomes, especially if issues of tolerance or acceptability arise in a subset of participants. Nevertheless, this design is well suited to exploratory research contexts where early validation of safety and feasibility is critical before advancing to broader investigations. Finally, the recruitment process may be subject to selection bias, as participation was based on patient willingness to volunteer.

### Future direction of the VIRAS project and perspectives for the use of VR in the operating room

Given the encouraging results obtained in this initial cohort and based on the sequential analysis design (SPRT), it has been decided to maintain the same statistical hypotheses for the second phase of the VIRAS project, which will involve patients undergoing awake brain surgery. The only modification concerns the maximum sample size, which will be limited to 25 participants due to recruitment feasibility. This second phase will begin promptly, using the same equipment and procedures as in the orthopedic cohort. An additional element will be the integration of a dedicated software application for the selection and control of neurofunctional tests. This application relies on an algorithm that incorporates both lesion laterality and tumor location, enabling dynamic adaptation of the test sequence. The proposed test battery was defined by our team based on the most recent literature, and the software faithfully reproduces our current practices while streamlining test selection and administration [35].

Beyond the technical validation, this initial phase has highlighted the importance of addressing anxiety related to the operating room environment for both patients and healthcare providers, especially during procedures performed under regional anesthesia. The results support the potential of VR not only for enhancing intraoperative neurofunctional monitoring, but also for providing psychological support. This potential could be extended to broader applications such as immersive preoperative operating room tours or the use of VR-assisted hypnosis to support regional anesthesia procedures. These developments are particularly relevant in the context of reducing reliance on general anesthesia, especially in orthopedic surgery when regional anesthesia is a viable option [27,28]. Future studies are needed to explore these avenues more comprehensively and to evaluate their impact on perioperative outcomes and patient experience [36,37].

## Conclusion

VR in awake craniotomy remains a novel and insufficiently studied approach, with only a few single-center publications and a high degree of methodological heterogeneity [23]. The VIRAS protocol was specifically designed to address these gaps by introducing standardized cognitive tasks validated in awake neurosurgery, and by using established assessment scales to evaluate the intraoperative tolerance of VR.

This first phase of the VIRAS study, conducted in the context of orthopedic surgery under regional anesthesia, confirms the excellent tolerance and feasibility of using a VR headset for intraoperative neurofunctional testing. The device was well accepted by patients and allowed complete testing without adverse events, while contributing to reduced anxiety and discomfort in the operating room.

The results also underscore the importance of involving all healthcare professionals early in the implementation of such innovative tools. The sequential analysis methodology used in this phase proved effective and now supports the transition to the next phase of the project, which will focus on awake brain surgery.

## Supporting information

S1 File Neuropsychological tests proposed by the VIRAS application. (DOCX)
