# Supplementary material for: Virtual Reality in Awake brain Surgery (VIRAS) stage I: Proof of concept and tolerance validation during scheduled orthopedic surgery
Source: PLoS One. 2025 Sep 3;20(9):e0329894. doi: 10.1371/journal.pone.0329894 (PMC12407469; doi:10.1371/journal.pone.0329894)
Supplement: S1 File — (DOCX) [file pone.0329894.s001.docx]

**Neuropsychological tests proposed by the VIRAS application**

- **Picture-naming task : DO 80** (Almairac F, Herbet G, Moritz-Gasser S, de Champfleur NM, Duffau H. The left inferior fronto-occipital fasciculus subserves language semantics: a multilevel lesion study. Brain Struct Funct. 2015 Jul;220(4):1983-95. doi: 10.1007/s00429-014-0773-1. Epub 2014 Apr 18. PMID: 24744151.)
- **DO 80 in quadrant** (Gras-Combe G, Moritz-Gasser S, Herbet G, Duffau H. Intraoperative subcortical electrical mapping of optic radiations in awake surgery for glioma involving visual pathways. J Neurosurg. 2012 Sep;117(3):466-73. doi: 10.3171/2012.6.JNS111981. Epub 2012 Jul 13. PMID: 22794319)
- **DO 80 N-1** (Puglisi G, Sciortino T, Rossi M, Leonetti A, Fornia L, Conti Nibali M, Casarotti A, Pessina F, Riva M, Cerri G, Bello L. Preserving executive functions in nondominant frontal lobe glioma surgery: an intraoperative tool. J Neurosurg. 2018 Sep 28;131(2):474-480. doi: 10.3171/2018.4.JNS18393. PMID: 30265193.)
- **Regulated words non-infrequent words and reg-ireg-non words frequent words** (Ruis C. Monitoring cognition during awake brain surgery in adults: A systematic review. J Clin Exp Neuropsychol. 2018 Dec;40(10):1081-1104. doi: 10.1080/13803395.2018.1469602. Epub 2018 Aug 1. PMID: 30067443).
- **PPTT** (Chang WH, Pei YC, Wei KC, Chao YP, Chen MH, Yeh HA, Jaw FS, Chen PY. Intraoperative linguistic performance during awake brain surgery predicts postoperative linguistic deficits. J Neurooncol. 2018 Aug;139(1):215-223. doi: 10.1007/s11060-018-2863-z. Epub 2018 Apr 10. PMID: 29637508; PMCID: PMC6061224 ; Ruis C. Monitoring cognition during awake brain surgery in adults: A systematic review. J Clin Exp Neuropsychol. 2018 Dec;40(10):1081-1104. doi: 10.1080/13803395.2018.1469602. Epub 2018 Aug 1. PMID: 30067443).
- **MT 86 picture** (Ruis C. Monitoring cognition during awake brain surgery in adults: A systematic review. J Clin Exp Neuropsychol. 2018 Dec;40(10):1081-1104. doi: 10.1080/13803395.2018.1469602. Epub 2018 Aug 1. PMID: 30067443).
- **RMET** (Prevost M, Carrier ME, Chowne G, Zelkowitz P, Joseph L, Gold I. The Reading the Mind in the Eyes test: validation of a French version and exploration of cultural variations in a multi-ethnic city. Cogn Neuropsychiatry. 2014;19(3):189-204. doi: 10.1080/13546805.2013.823859. Epub 2013 Aug 13. PMID: 23937473. ; Yordanova YN, Duffau H, Herbet G. Neural pathways subserving face-based mentalizing. Brain Struct Funct. 2017 Sep;222(7):3087-3105. doi: 10.1007/s00429-017-1388-0. Epub 2017 Feb 27. PMID: 28243761.)
- **VOSP** (Lezak, M. D., Howieson D. B., Loring D. W., Hannay H. J. & Fischer J. S. (2004) neuropsychological assessment, 4th Ed. Oxford : Oxford University Press.)
- **PEGV** (Agniel, A., Joanette, Y., Doyon, B., & Duchein, C. (1992). Protocole MontréalToulouse d'évaluation des gnosies visuelles. France: L'Ortho-Édition.)
- **STROOP** (Puglisi G, Sciortino T, Rossi M, Leonetti A, Fornia L, Conti Nibali M, Casarotti A, Pessina F, Riva M, Cerri G, Bello L. Preserving executive functions in nondominant frontal lobe glioma surgery: an intraoperative tool. J Neurosurg. 2018 Sep 28;131(2):474-480. doi: 10.3171/2018.4.JNS18393. PMID: 30265193.)
- **Reading and comparing numbers, calculation** (Ruis C. Monitoring cognition during awake brain surgery in adults: A systematic review. J Clin Exp Neuropsychol. 2018 Dec;40(10):1081-1104. doi: 10.1080/13803395.2018.1469602. Epub 2018 Aug 1. PMID: 30067443)
